# Decorin Content and Near Infrared Spectroscopy Analysis of Dried Collagenous Biomaterial Samples

**DOI:** 10.3390/biom2040622

**Published:** 2012-12-14

**Authors:** Mila L. Aldema-Ramos, Joan Carles Castell, Zerlina E. Muir, Jose Maria Adzet, Rosa Sabe, Suzanne Schreyer

**Affiliations:** 1 U.S. Department of Agriculture, Agricultural Research Service, Eastern Regional Research Center, 600 E. Mermaid LN, Wyndmoor, PA 19038, USA; E-Mail: zerlina.muir@ars.usda.gov (Z.E.M.); 2 Asociación de Investigación de la Industria de Curtidos y Anexas (AIICA). Pla de la Massa, s/n, Igualada, Spain; E-Mails: joanccastell@gmail.com (J.C.C.); info@aiica.com (J.M.A.); innovation@aiica.com (R.S.); 3 Chemometrics Applications, Thermo Fisher Scientific, Tewksbury, MA 01876, USA; E-Mail: suzanne.schreyer@thermofisher.com (S.S.)

**Keywords:** proteoglycan, decorin, sulfated glycosaminoglycan (sGAG), Alcian blue, near infrared, principal component analysis (PCA), leather quality

## Abstract

The efficient removal of proteoglycans, such as decorin, from the hide when processing it to leather by traditional means is generally acceptable and beneficial for leather quality, especially for softness and flexibility. A patented waterless or acetone dehydration method that can generate a product similar to leather called Dried Collagenous Biomaterial (known as BCD) was developed but has no effect on decorin removal efficiency. The Alcian Blue colorimetric technique was used to assay the sulfated glycosaminoglycan (sGAG) portion of decorin. The corresponding residual decorin content was correlated to the mechanical properties of the BCD samples and was comparable to the control leather made traditionally. The waterless dehydration and instantaneous chrome tanning process is a good eco-friendly alternative to transforming hides to leather because no additional effects were observed after examination using NIR spectroscopy and additional chemometric analysis.

## 1. Introduction

Hides are the most important co-product of the meat industry. They are a $2.2 and €3.4 billion export commodity for the USA and EU, respectively. Leather is the most valuable commodity produced from hides. The current research project deals with the necessity of reducing the burden that traditional tanning of hides to leather impose on the environment by implementing a waterless tanning or dehydration method that can generate a product called Dried Collagenous Biomaterial (known as BCD). The Spanish collaborators conducted the previous studies and prepared the BCD samples at the Association of leather and Related Products Research *Asociación de Investigación de la Industria de Curtidos y Anexas (AIICA*) facility in Igualada, Spain. A patented dehydration method (international patent number PCT / IB2009 / 055733; December, 2009) [1] based on acetone drying and an instantaneous chrome tanning process was utilized [2,3]. AIICA scientists have researched the development of a method that improves the quality and durability of the BCD materials in producing the crust leather with improved mechanical properties which are comparable to traditionally tanned leather [2,3]. The physical properties of BCD are comparable to leather because it is stabilized, non-putrescible, opaque and a flexible material. However, BCD is not “tanned”, it can be further stabilized by the “instantaneous tanning process” with chrome salts or vegetable extracts. The properties of BCD allow for very fast tanning reactions by immersion of the BCD pelts, therefore, wet blue or vegetable leather can also be obtained from BCD [2,3]. 

**Figure 1:**
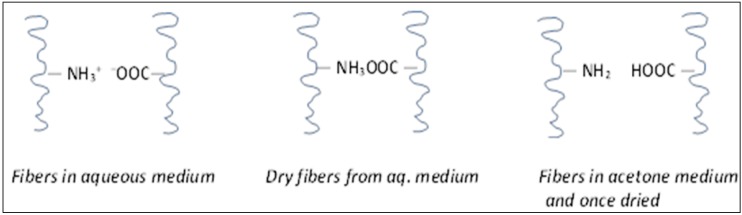
Reactive group charges in different medium.

A translucent material with a corn-like structure is obtained when a delimed and bated pelt is air dried (dry fibers). It is tough and compact and quite hard to penetrate by any solvent. In order to obtain a spongy, flexible and opaque material that has strong solvent absorption capacity, the fibers must remain separated during the drying process [2,3]. One way to accomplish this is to change the medium polarity from water to a less polar organic solvent such as acetone as depicted in Figure 1. 

By reducing the immersion medium polarity, the ionic groups of the fibers are discharged. However, drying pelt in aqueous medium, the groups are linked together by electrostatic forces (as they appear in the central Figure). If the acetone is removed afterwards, the reactive groups are devoid of the electrical charges. During the drying operation, they cannot link to each other and the fibers remain separated resulting to a fibrous pelt that is flexible, opaque, white and spongy [1,2,3]. Decorin is a small extracellular matrix proteoglycan involved in several fundamental biological functions, including “decorating” or as a glue in the organization of collagen fibrils in animal skin [4,5]. A decorin molecule consists of a core-protein and a carbohydrate side chain, glycosaminoglycan, or dermatan sulfate (sGAG). Depending on tissue and species, the core protein has a molecular weight of about 40 kDa and the dermatan side chain has a molecular weight that varies between 20–50 kDa [4,5]. In nature, the Leucine-rich repeats (LRR) segments of the core decorin protein adopt a stacked β-sheet α-helix hairpin structure resulting in an overall horseshoe shape structure that tends to dimerize as its stable configuration [4,5]. The effects of decorin removal on the ultimate properties of the leather made from the differently treated hides were previously determined. ARS scientists previously established that as the decorin content is further diminished, the leather product became softer, more stretchable, at the same time tougher than the control leather tanned traditionally [6,7]. AIICA and ARS researchers are particularly interested in the relationship of the available residual decorin content to the properties of leather product obtained from the BCD materials. The proteoglycan decorin content analysis is based on the assay of its glycan or sulphated glycosaminoglycan (sGAG) portion [7,12]. The principle is based on the specific interaction between negatively charged polymers such as sGAG and a positively charged cationic dye. The cationic dye is Alcian blue, a tetravalent cation with a hydrophobic core [7,10,12]. The positively charged dye binds to the negatively charged polymers such as sGAG at high ionic strength. This dye binds more tightly to sGAG than monovalent cationic dyes. The ionic bonding between cationic dyes (such as Alcian blue) and the negatively charged sGAG are generally thought to be proportional to the number of negative charges present on the sGAG chain, *i.e*., both sulfate and carboxyl groups [7,10,12]. The intensity of absorption of the bluish coloration is directly proportional to the sGAG concentration in the sample.

## 2. Results and Discussion

### 2.1. Acetone Drying Efficiency

To measure the drying efficiency of acetone on BCD samples, the water content in the acetone washing floats was monitored and analyzed by Karl-Fischer technique [1,2]. The amount of water removed (reported here as averages of two trials) from the sample was about ~24.5% after just one acetone drying/washing (sample 1S) as shown in Table 1. The N/A comment for the untreated sample 5S, meant that the water content could not be determined because there was no acetone washing float available. After the second acetone drying step (sample 1S), the washing float contained ~7% water, whereas after three acetone washes, the water content was ~3% for sample 2S. But after fourth (sample 3S) and fifth (sample 4S) acetone washes, the amount of water removed plateaued at ~1.5 %, implying the low water content remaining in the sample. Only samples 3S and 4S could be well chrome tanned to good quality crust leather products giving BCD materials that were flexible and soft [2,3]. Sample 0S (1 acetone wash) was not included in further experiments for analysis and characterization due to its high water content and not considered a BCD sample. 

**Table 1:** Water content analysis in acetone washings.

Sample code	Sample washing / drying tretament	Water content in acetone washing float (%)
0S	Acetone dehydrated / 1 wash within 60 min	24.5 ± 0.10
1S	Acetone dehydrated / 2 wash within 60 min	6.8 ± 0.15
2S	Acetone dehydrated / 3 wash within 60 min	3.0 ± 0.00
3S	Acetone dehydrated / 4 wash within 60 min	1.8 ± 0.05
4S	Acetone dehydrated / 5 wash within 60 min	1.3 ± 0.00
5S	No Acetone dehydrated / Air dried	N/A

### 2.2. Decorin Analysis Based on SGAG Portion of the Molecule

The established method utilizing Alcian Blue colorimetric determination of decorin based on the sGAG portion of the decorin molecule was utilized [7,10]. An assay technique was adapted from the sGAG Assay Kit which was originally designed for liquid samples [12]. It was developed and made compatible to the BCD materials capable of generating reliable data. 

**Figure 2:**
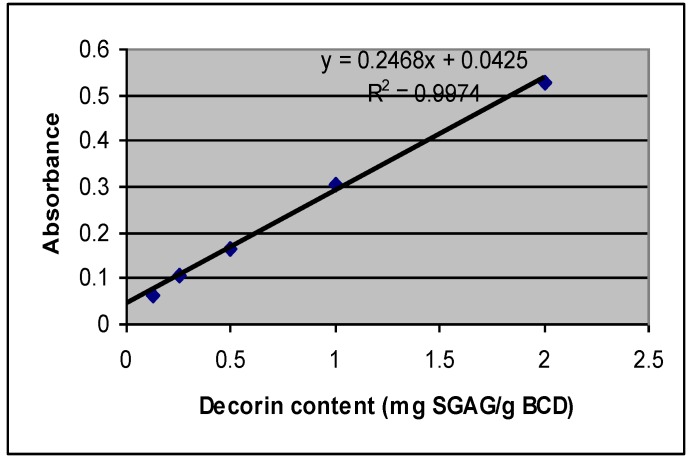
Standard graph of Decorin, based on known sulfated glycosaminoglycan (SGAG) concentration.

The concentration of decorin in BCD samples, with respect to its sGAG content, was calculated from the slope of the standard calibration graph (Figure 2). The standard graph is prepared by plotting a straight line relating the absorbance to the known amount of standard sGAG. Regression equations are established as standard curve describing the relationship between the intensity of visible light absorption to the known concentration of standard decorin with Alcian Blue dye [7,10,12]. 

The modified Alcian Blue assay has improved efficiency, capable of analyzing more samples and requiring lesser amounts of reagents because small aliquot amounts from each sample were utilized [6,7]. The core protein of proteoglycans may be degraded by proteolytic activity in the sample which, however, does not alter the sGAG chains. 

**Figure 3:**
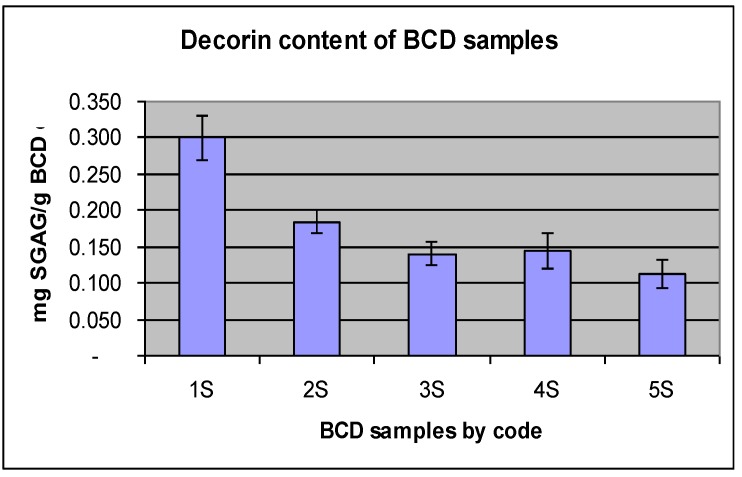
The decorin content of differently acetone dried bovine Dried Collagenous Biomaterial (BCD) samples.

The amount of decorin removed reached an optimum value after fourth acetone washes (3S) because no additional decorin was further removed in the fifth wash (4S) as shown in Figure 3. The amount of decorin available in BCD samples after fourth washes was about 1.4 mg/g BCD and relatively comparable to traditionally tanned crust leather. The decorin content of the wet blue obtained from traditionally tanned hide, was found to have an average of ~1.6 mg SGAG/g lyophilized hide when analyzed in parallel with BCD samples. This implied that the decorin removal by acetone drying process was relatively comparable in efficiency to the traditional tanning treatments.

### 2.3. Mechanical Properties of BCD Samples

Results of the research are based on softness performance and measurements of tensile strength and elongation-to-break. The 2,500 Newton and 562 lbf (pound force) load cell was used for the mechanical property testing of the BCD samples because they were harder than the traditionally tanned leather products. The results of the mechanical property measurements and the amount of available decorin in BCD samples are shown in Table 2. 

**Table 2:** Correlation of decorin content to the mechanical properties of the BCD samples.

Sample Code	Sample Acetonedrying Treatment	Elongation-to-break, %	Tensile Strength, N/m²	mgSGAG/g BCD
**1S**	Acetone dehydrated /2 washes	67	386	0.30 ± 0.07
**2S**	Acetone dehydrated /3 washes	68	369	0.18 ± 0.04
**3S**	Acetone dehydrated /4 washes	86	405	0.14 ± 0.05
**4S**	Acetone dehydrated /5 washes	76	361	0.14 ± 0.06

**Figure 4:**
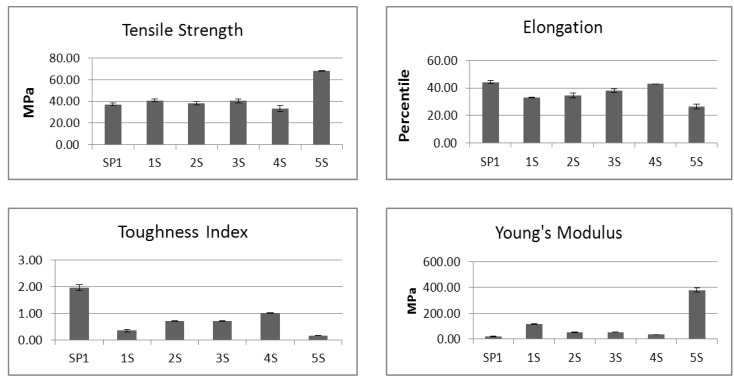
The graphical presentation of the mechanical properties of BCD samples. The sample code and the number of acetone washes are the same as in Table 1 and Table 2.

The following bar graphs in Figure 4 show how the mechanical properties are correlated to the number of acetone washes of the different BCD samples. 

As shown in Figure 4, the BCD samples dried with acetone four to five times (3S and 4S) were softer and more elongated than those untreated (5S) or acetone dried only 1 time (2S). SP1 was another BCD sample from different bovine hide that was previously washed five times with acetone (comparable to sample 4S) and both behaved similarly. SP1 was analyzed and found to have about 0.13 mg SGAG/g BCD, close to the amount found in 4S with about 0.14 mg SGAG/g BCD. The BCD samples dried/washed four to five times with acetone exhibited the best quality with relatively high toughness index, high elongation and low Young’s modulus (or softer). The untreated sample 5S appeared stiff and quite heavy and required only a small amount of sample to give the same weight as the acetone treated samples 1S to 4S. The amount of decorin available in 5S was found to be 0.11 mg/g, was based on a gram of that sample and not in direct comparison to the decorin content per gram of the BCD samples which appeared to be lighter and required more material and in turn resulted in a slightly higher concentration of decorin content per g weight of the sample. It was also quite difficult to measure the mechanical properties of the untreated (5S) sample due to its stiff and brittle nature. 

### 2.4. Near Infrared (NIR) Spectroscopy

The goal was to find out if there was any structural changes that took place in the sample based on the NIR spectra of the differently treated BCD samples [13,14]. A total of 18 spectra were taken for each sample: nine for the inside or corium side and nine for the outside or the grain side of the hide samples. Samples labeled 1S to 5S, including SP1, were used/analyzed as received. Sample 5S was a stiff air dried rawhide untreated with acetone and was distinct from the other samples. The spectra were separated into inside and outside groups and averaged over all positions and replicated to generate average spectra for 1S to 5S on each side. 

**Figure 5:**
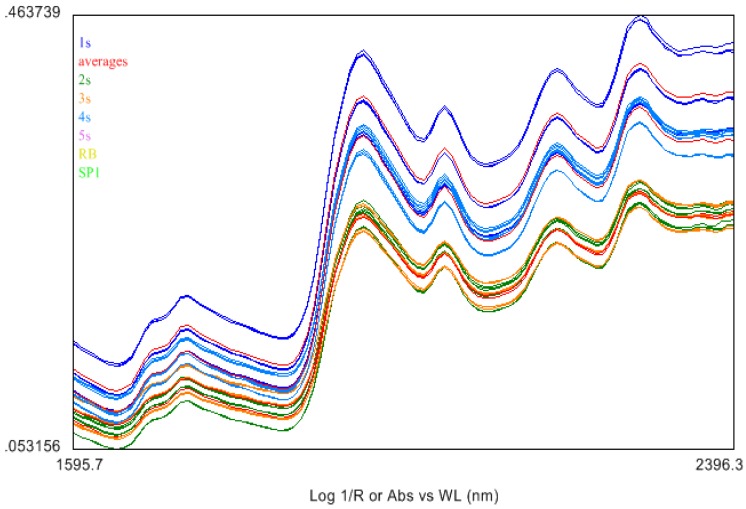
Near Infrared (NIR) spectra of different BCD (outside or grain) samples. Plot shows absorbance as Log(1/R) at each wavelength.

**Figure 6:**
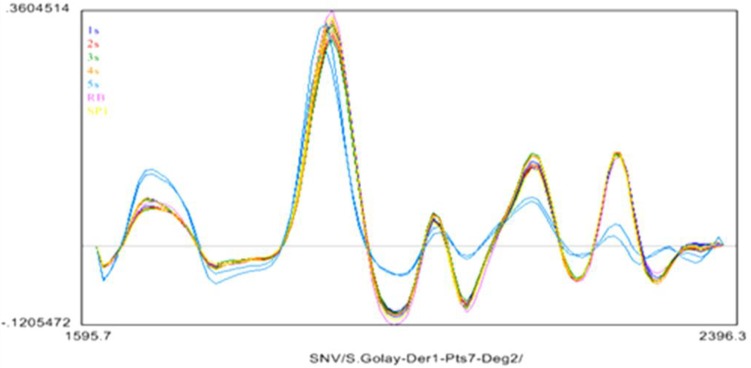
Preprocessed (SNV, Savitsky-Golay (1-7-2)) NIR spectra of the samples. Plot shows the preprocessed absorbances at each wavelength.

The NIR spectral features of the acetone dried BCD samples looked similar to those of traditionally tanned leather products [17]. Spectra were preprocessed by using an internal software package. Savitsky-Golay preprocessing (first derivative, seven point smoothing, second order polynomial fit) was performed on the NIR spectra to smoothen the data and remove the noise as shown in Figure 6. The Standard Normal Variate (SNV), normally performed to offset particle size/packing differences of the different samples was done to compensate for differences in roughness of the hide samples at various positions. 

There were no distinct spectral features to differentiate samples 1S to 4S whereas the untreated sample 5S (light blue spectra) exhibited peak shifts and height differences compared to treated samples 1S to 4S. Larger variance was observed in the individual 5S spectra, and was also noisier than the other samples. Because of the high signal to noise ratio in NIR instruments, derivatives are frequently used for identification and quantification using these bands as shown in Figure 6.

**Figure 7:**
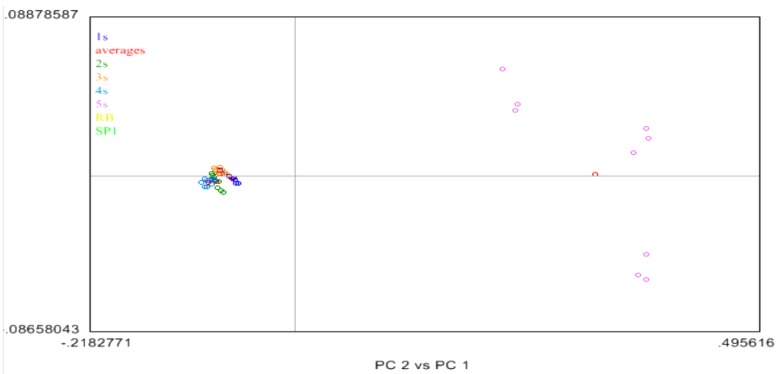
Principal component analysis (PCA) of the NIR data of 1S-to-5S and SP1 BCD samples in the hide grain.

PCA plots were generated for the grain or the outside spectra of the different samples [13,16,17]. The clustering of samples based on similar structures found on the respective sides analyzed was generated based on the PCA plots of samples 1S to 5S, and SP1Sample 5S (in purple) had unique physical properties compared to the other five BCD samples that the PCA plots could consider as an anomalous sample (purple separated dots) as shown in the following Figure 7. It is postulated that these differences are due to the differences in the moisture of the samples, which in turn results in changes in the physical structure observed in 5S. A further look at the loadings associated with PC1 and PC2 indicated both the water band (1950 nm) and the CH overtone bands (2130–2300 nm) showed features important to PC1 and PC2. Due to the general nature of NIR, it is difficult to associate any further assessment of any specific regions that are causing the differences associated with 5S and the rest of the samples. However PC plots up to 4 PCs did not show any significant differences from that observed in PC 1 and PC2. In all these cases, there was a unique cluster for samples 1S through 4S, and then a unique cluster for 5S. Again examination of the loadings for these PCs showed features relating to both the water peak and the CH region from 2100–2300 nm.

**Figure 8:**
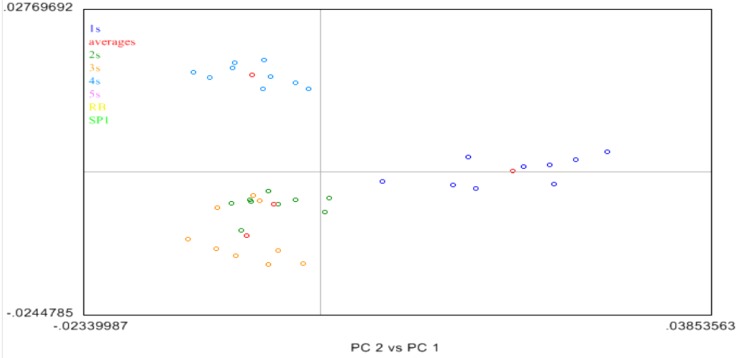
Principal component analysis (PCA) of the NIR data of 1S to 4S BCD samples.

The PCA analysis [14,16] of the acetone treated samples 1S to 4S appeared closely related to each other as they form one cluster in the left 2 quadrants of Figure 7. Next, sample 5S was removed and the remaining 1S to 4S samples were analyzed by PCA. In this case three (3) distinct clusters were observed as shown in Figure 8. Sample 1S (purple) and, sample 4S (blue) were in unique clusters, albeit the variance associated with these clusters is quite large. However samples 2S (orange) and 3S (green) clustered together and were not differentiable from each other. The average of each sample spectra was shown as a red dot. In these cases, while slight differences are observed between Samples 1S and 2S, these differences are small on the scale of the PC plot, and due to the large variation observed in these clusters, the differences are not robust.

The same analysis was done to the NIR spectra of the inside (or corium) portion of the BCD samples. The same behavior was exhibited when the NIR spectra of the corium of the BCD samples were taken and analyzed. The only distinct cluster was again due to the untreated sample 5S (purple), showing a large spread in the cluster with high variance. Other samples (1S to 4S, blue, green, orange, and yellow) were in one cluster, although they tend to be part of tight grouping by a sample in that super-group. 

## 3. Experimental Section

### 3.1. Materials and Method

The SGAG assay Kit was obtained from Kamiya Biochemicals Inc. [12]. Hydroxylamine hydrochloride and sodium hydroxide from Sigma-Aldrich (St. Louis, MO, USA), Boron TS and Rohapon 6000 from TFL USA/Canada (Greensboro, NC, USA). Proxel from Chemtan Co. (Exeter, NH, USA), Protease inhibitor cocktail for mammalian tissues, #P-8340: Sigma. Guanidine hydrochloride (GuHCl): Mallinckrodt #7716, Bio-reagent grade, Thomas Scientific (Swedesboro, NJ, USA). The Microplate reader used was a Multiskan/MCC340 from Thermo Labsystems (or any spectrophotometer with 600–620 nm filter will do). Precision pipettes with disposable tips. Disposable syringes with 18 G needle were used for convenient removal of supernatants. Capped polypropylene vials (1.5 or 2 mL size) of Eppendorf type are recommended. Centrifuge capable of giving a centrifugal force of at least 12,000× g is required.

### 3.2. Preparation of Dried Collagenous Biomaterial (BCD)

**Figure 9:**
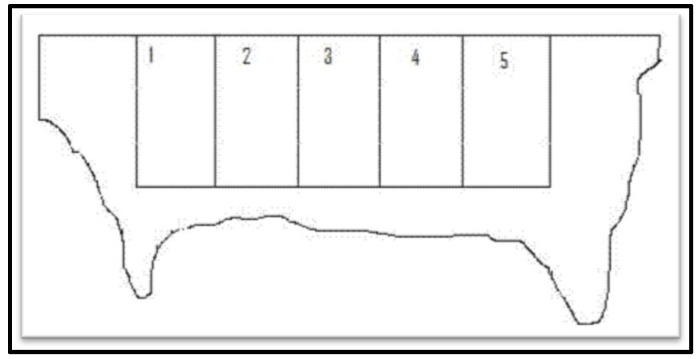
How five strips of hides were taken for further experimentation.

One half of a bovine hide sample was treated traditionally from dehairing to deliming stage [2,6,7]. The hide was pickled to pH 3.5 with ~1.5% formic acid then washed two times with 200% (w/w) water for 20 min [2,3]. In the pickle stage, the side was sammed and cut into five strips as indicated in Figure 9. 

Each strip was identified with a different number of holes. The pickled strips were neutralized for 3h with ~1% sodium bicarbonate to ~pH 5, then washed twice with 200% water, and mechanical sammed before acetone dehydration treatment [2,3]. To monitor the water removal from the hide, the water content in the residual acetone washing floats were analyzed by Karl-Fischer technique. The five (5) hide pieces were treated differently with varying number of acetone washes at 200% (W/W) float. The acetone washes were performed on each neutralized pickled hide strip samples to convert them to BCD samples [2,3]. 

### 3.3. Sample Preparation

#### 3.3.1. Pulverization of BCD Samples

An adaptation of the protocol previously developed for the pulverized hide pieces was used in the subsequent steps in the tanning process [7,10]. The BCD samples were first cut to small square pieces (about 5–10 mm X 5–10 mm) with a sterilized/cleaned pair of scissors. About 4 g of cut pieces was weighed into a wide mouth glass vial with plastic top and moistened by adding ~80% (w/w) water and mixing well. The moistened samples were frozen for at least 16 h. The samples were pulverized in a cryogenic mill (6800 Freezer Mill, SPEX CertiPrep, Metuchen, NJ, USA). The pulverized samples were lyophilized overnight (~20 h) in a freeze dryer under vacuum using Virtis Sentry 2-0, Model: Freeze Mobile 25ZS vacuum drier.

### 3.4. Extraction of the Proteoglycan, Decorin, from Powdered BCD Samples

The assay is designed to detect sGAG in biological samples such as synovial fluid, blood, and tissue extracts [10,12]. Since the BCD sample is solid, extraction of the proteoglycan with 4M Guanidinium Hydrochloride (GuHCl) was performed first [6,7]. About 50 mg of the powdered and lyophilized BCD samples were weighed into a 1.5 mL eppendorf tubes. Three trials were performed for each sample. Protein was extracted with 0.75 mL Ca Tris Buffer (pH 6.8) and 0.5 mL of 8 M guanidine-HCl in phosphate buffer at pH7.6. Collagenase (20 U) was added to remove unwanted matrix interferences [6,7]. Protease inhibitor (20 µM) cocktail (25 µL of the 1.0 mM cocktail was added in 1.3 mL final volume of the assay solution) was added to protect the protein from possible proteolytic degradation from proteases present in the solution [6,7]. The resulting mixture was mixed well by shaking at 27 °C for 10 min in water bath, stirred overnight in a rotatory mixer/gyrator or spinner, and centrifuged the following day. Cell debris and insoluble material should be removed by centrifugation at ~12,000× g for 15 min. The insoluble precipitate, including sample debris, was discarded. 

### 3.5. Decorin Analysis Based on sGAG Portion of the Molecule

The Alcian Blue stock solution (3 mg/mL) containing 0.1% H_2_SO_4_ and 0.4 M GuHCl was prepared [7,10,12]. The final concentration of Alcian blue used in each assay solution was about 0.15 mg/mL. Aliquots of 8M GuHCl was used to dilute the samples to final concentration of 0.4 M in the assay solution. The solution used in diluting the Alcian Blue stock solution and as addition to samples contained 0.3% H_2_SO_4_ and 0.75% Triton X-100. The DMSO solution used in washing off contaminations and interfering matrices in pellets was composed of ~ 40% dimethylsulphoxide and 0.05 M MgCl_2_. Gu-Prop solution containing 4 M GuHCl, 33% 1-propanol and 0.25% Triton X-100 is used to dissolve bluish pellets before reading the absorbance [7,10,12]. 

For calibration purposes, the set of known concentration of sGAG, ranging from 5–100 µg/mL that was provided with the Kit was analyzed by taking absorbance readings at 605 nm against the reagent blank in order to generate a standard graph [7,10,12]. The graph was prepared by plotting a straight line relating the absorbance to the known amount of standard sGAG. The concentration of decorin in BCD samples with respect to its sGAG content, were calculated from the slope of the standard calibration graph of Figure 2.

### 3.6. Determination of Mechanical Properties

Mechanical property measurements of the BCD samples included tensile strength (or “toughness”) which is measured in units of force per unit area such as (megapascal, MPa or Newton per meter square (N/m²), elongation-to-break, is the measure of “strechability” (in percentile), Young’s modulus (in MPa) (measure of “stiffness”). Five dog bone-shaped leather samples (1-cm × 10-cm) were cut near the standard test area as described in ASTM D2813-03 [11] with the long dimension parallel to the backbone. The average thickness of the leather samples varied from 1.7 mm to 2.7 mm. An upgraded Instron mechanical property tester, model 1122 (Instron, Norwood, MA, USA), and Testworks 4 data acquisition software (MTS Systems Corp., Minneapolis, MN, USA) were used throughout this work. The strain rate was set to 25.4 cm/min with a grip distance of 5 cm. Each test was conducted on five samples to obtain an average value [11].

### 3.7. The Near Infrared Spectroscopy

The chemical structure at the surface of the BCD sample was analyzed by utilizing near InfraRed (NIR) Spectroscopy. The NIR signals are due to the excitation of vibrational modes within molecules where the strong and significant signals correspond to functional groups with dipole moments containing NH, OH or CH bonds [13,14]. The NIR measurements do not require rigorous sample preparation, especially with the robust handheld NIR instrument such as Phazir from Thermo Fisher Scientific, and still capable of giving valuable information [15]. To determine the differences and/or similarities of the differently treated BCD samples, the Principal Component Analysis (PCA) [16] were performed on the NIR spectra of each sample. PCA is a statistical calculation used in extracting the systematic variations in the data. 

## 4. Conclusions

The physical appearance and characteristics of the samples that were dehydrated three to five times by acetone BCD (samples 2S, 3S and 4S, respectively) were similar. But only after four acetone treatments was a good quality BCD sample obtained. An optimum efficiency in decorin removal was attained after four acetone washings because no further decorin removal was observed in the sample washed five times with acetone. Both BCD samples possessed a soft, opaque and firm crust leather texture. The NIR spectra of the acetone-treated BCD samples were only slightly differentiable in the PCA cluster plots, or according to sample grouping in the statistical analysis. The broad bands in NIR spectra could arise from the overlapping absorption bands of polar functional groups in different intramolecular and extramolecular environments of the molecules in the sample. Overall, the analysis of inside (corium) *vs*. outside (grain) of the BCD samples generated similar results. The spectral features were super-imposable and not distinguishable from each other. On the other hand, the air dried BCD sample (5S) appeared translucent and very hard material and its NIR spectra were distinct from the other four acetone dried samples. This implied that the waterless drying treatment before chrome tanning was quite resilient to any harmful effects of the organic solvent. The mechanical properties or quality of BCD samples washed/dried with acetone four to five times were comparable to the traditionally tanned crust leather products. The acetone dehydration technique was quite superior to the traditional tanning process because almost no waste water was generated by this technique. The process could also be quite economical because the acetone washes can be collected, recovered and recycled.
